# Osteoporosis and fragility fracture burden in Asia Pacific: Key findings from the APCO-IOF Asia Pacific Regional Audit

**DOI:** 10.1016/j.afos.2026.05.004

**Published:** 2026-06-06

**Authors:** Hui Min Charlotte Choo, Paul Mitchell, Peter R. Ebeling, Philippe Halbout, Nicholas C. Harvey, Eugene V. McCloskey, Ambrish Mithal, Manju Chandran

**Affiliations:** a Osteoporosis and Bone Metabolism Unit, Department of Endocrinology, Singapore General Hospital, Singapore, Singapore; b University of Notre Dame Australia, Sydney, Australia; c Synthesis Medical NZ Limited, Dunedin, New Zealand; d Department of Medicine, School of Clinical Sciences, Monash University, Clayton, Victoria, Australia; e Department of Endocrinology, Monash Health, Clayton, Victoria, Australia; f International Osteoporosis Foundation, Nyon, Switzerland; g MRC Lifecourse Epidemiology Centre, University of Southampton, Southampton General Hospital, Southampton, UK; h NIHR Southampton Biomedical Research Centre, University of Southampton and University Hospital NHS Foundation Trust, Southampton, UK; i Mellanby Centre For Musculoskeletal Research, University of Sheffield, Sheffield, UK; j Centre for Metabolic Diseases, University of Sheffield Medical School, Sheffield, UK; k Division of Clinical Medicine, School of Medicine & Population Health, University of Sheffield, Sheffield, UK; l Institute of Endocrinology and Diabetes, Max Healthcare, New Delhi, India; m DUKE NUS Medical School, Singapore, Singapore

**Keywords:** Asia Pacific, Audit, Fracture, Osteoporosis, APCO, IOF

## Abstract

Rapid population aging in the Asia Pacific region will significantly increase its burden of osteoporosis and fragility fractures. The Asia Pacific Consortium on Osteoporosis (APCO)-International Osteoporosis Foundation (IOF) Asia Pacific Regional Audit was developed through a coordinated effort of these two organizations. Spanning data from 22 countries and regions in the Asia Pacific, it comprehensively assembles the most current regionally relevant osteoporosis evidence available. The audit used a structured questionnaire covering 12 domains, supplemented by demographic projections, registry data, government statistics, and expert consensus estimates where formal data were unavailable. The audit revealed that osteoporosis is a national health priority in only six countries, limiting attention, funding, and coordinated service development. It also highlights glaring infrastructural gaps: insufficient fracture surveillance, uneven diagnostic access, misaligned reimbursement, and limited secondary fracture prevention. Collectively, these deficiencies point not to a lack of evidence, but to an implementation deficit in translating evidence into routine care. The audit proposes several key solutions including implementing fracture registries and post-fracture care pathways, developing coherent country-specific guidelines, aligning reimbursement with risk profile, and tracking quality outcomes. These findings provide a timely roadmap for governments and health care systems to reduce fracture burden and improve equitable osteoporosis care across the Asia Pacific region.

## 1 Introduction

Over the coming decades, the Asia Pacific region will experience drastic demographic transformation. By 2075, the population will grow from 4.24 to 4.45 billion, and the region will be home to almost half the world's population [1]. It is predicted that the average life expectancy of individuals living in the Asia Pacific will rise by almost a decade from 78 to 87 years, and up to one-third of the population will be aged 70 years or older in several countries by 2075 [1]. These population dynamics matter profoundly for osteoporosis because fracture risk rises significantly with age [2]. Hip, vertebral, humeral, and distal forearm fractures are rarely isolated events; rather, they are associated with recurrent fractures, chronic pain, disability, loss of independence, caregiver burden, and escalating downstream costs [3]. This will pose immense strain on the healthcare and social infrastructure if anticipatory systematic changes are not put in place.

Recognizing this potential crisis, the Asia Pacific Consortium on Osteoporosis (APCO), in collaboration with the International Osteoporosis Foundation (IOF), performed an audit in 22 countries and regions in the Asia Pacific, to understand the epidemiology, healthcare infrastructure, policy frameworks, and management of osteoporosis in the region [4]. This audit reflects the substantial evolution in osteoporosis awareness, fracture prevention initiatives, and healthcare system preparedness across the Asia Pacific region since earlier IOF regional assessments, while simultaneously highlighting persistent and emerging gaps in care delivery [4]. This review summarizes the key findings of the APCO-IOF regional audit 2025.

## 2 Methodology

Multi-source data from individual specialists and osteoporosis professional societies was obtained via a standardized data questionnaire covering 12 domains. These domains encompassed key components of osteoporosis care systems, including centralized fracture database, clinical workforce involvement, patient support organizations, recognition of osteoporosis as national health priority, availability and reimbursement of medications, Fracture Liaison Services (FLS), hip fracture surgery wait times, national osteoporosis guidelines, diagnostic access, fracture risk assessment tools, and quality indicators. The 22 participating countries and regions were selected based on the availability of APCO representatives, collaboration with national professional societies linked to the IOF Committee of National Societies, and the availability of sufficiently robust country-level data across the audit domains. The countries and regions included in the Audit are Australia, Bangladesh, Bhutan, Brunei, China, Chinese Taipei, Hong Kong SAR, India, Indonesia, Japan, Malaysia, Mongolia, Myanmar, Nepal, New Zealand, Pakistan, Philippines, Republic of Korea, Singapore, Sri Lanka, Thailand, and Vietnam.

Data collection occurred between November 2023 and March 2024 through APCO representatives, national professional societies connected to the IOF Committee of National Societies, and the IOF Asia-Pacific University Network. Submitted data underwent central validation for internal consistency, with discrepancies clarified with country contributors. Data sources varied between countries depending on national data availability and included national registry data, published epidemiological studies, institutional datasets, government statistics, and expert consensus estimates where comprehensive nationwide data were unavailable. This was supplemented by statistical reports and demographic projections, providing an unprecedented overview of the eco system prevailing across countries. Demographic projections were derived from the U.S. Census Bureau International Database (IDB). Descriptive analyses were performed to generate country-specific and regional summaries. The audit authors subsequently undertook a final review in September 2025 to ensure that subsequent updates had been incorporated and that country summaries were contextually accurate. Readers seeking the full country-level audit data and detailed domain-specific analyses are referred to the primary APCO-IOF Asia Pacific Regional Audit publication and associated online resources [4].

## 3 Results

This review uses the audit as a regional policy and systems dataset, aiming to address the following questions: First, how quickly is the at-risk population growing? Second, how visible is the burden through registries and surveillance? Third, how prepared are systems in terms of policy, guidelines, workforce, and quality measurement? Fourth, how easily can individuals obtain diagnosis, risk stratification, surgery, and effective treatment? Fifth, where do examples of mature practice already exist, and what can be learned from them?

### 3.1 Recognition of osteoporosis as a public health concern

#### 3.1.1 The burden of osteoporosis

Osteoporosis is a systemic skeletal disease characterized by low bone mass, micro-architectural disruption and skeletal fragility, resulting in increased risk of fractures [5]. It is estimated that globally, one in three women and one in five men aged 50 years and older will sustain an osteoporotic fracture during their lifetime [6,7]. This pattern was found to be present in the Asia Pacific region also. Evidence from a systematic review of seven developed economies in the region (Australia, China, Hong Kong SAR, Japan, Singapore, Republic of Korea, and Chinese Taipei) revealed that osteoporosis prevalence ranges from 10 to 30% among women aged 40 years and older, and up to 10% among men, with fracture incidence typically between 500 and 1000 per 100,000 person-years in adults aged 50 years and above [8]. Comparable patterns were identified in less well-developed regional settings through the audit; for example, tertiary center-based data from Myanmar suggest that the prevalence of osteoporosis among postmenopausal women ranges from 35% to 45%.

The burden of osteoporosis is not simply the number of individuals with low bone mass and a fragility fracture [9]. It is the cumulative burden of first fractures, second fractures, delayed diagnosis, surgery that could have been avoided, long-term care, and preventable loss of independent living [10]. Hip fractures are particularly illustrative because they are clinically visible and economically costly [11]. They carry a 20-30% mortality risk within the first year, and survivors often experience long-term disability, loss of independence and increased financial burden. Costs arise not only from hospitalization and surgery, but also rehabilitation and long-term care [11]. Vertebral fractures, often silent are associated with significant morbidity [12,13]. Distal forearm and humeral fractures may appear less catastrophic, yet they are associated with reduced quality of life and are sentinel events that identify individuals at high future risk [3]. As populations age, healthcare expenditures related to osteoporosis are expected to increase significantly [14]. Health systems that treat each fracture as an isolated episode rather than as evidence of an underlying chronic disease will continue to incur avoidable recurrent harm.

#### 3.1.2 Demographic shift across the Asia Pacific region

In the next half a century, dramatic shifts in population dynamics are expected across the Asia Pacific region.

There are four broad categories of expected demographic trajectories (Table 1).

**Table 1:** Projected demographic trajectory categories across Asia Pacific countries and regions based on population projections (2025–2075) from the U.S. Census Bureau International Database [1].

	Trajectory		Countries and regions
1	Steady population growth from 2025 to 2075		Australia, Brunei Darussalam, Malaysia, New Zealand, Pakistan, Philippines
2	Growth till mid-century, followed by a plateau or slight decline		Bangladesh, Bhutan, India, Indonesia, Mongolia, Myanmar, Nepal, Singapore, Vietnam
3	Plateau by 2030, followed by accelerating population decline		China, Chinese Taipei, Hong Kong SAR, Japan, Republic of Korea, Thailand
4	Plateau from 2025 to 2050, followed by decline		Sri Lanka

Although these demographic patterns vary across countries, they converge in one critical respect: aging. Improved healthcare and living standards have also increased life expectancy worldwide. Together, older adults will represent a growing proportion of the population, and they indeed are the most vulnerable to osteoporosis and fragility fractures [15].

Amongst aging populations in the world, Japan ranks highest with more than 28% of its population aged 65 years or older. South Korea and Singapore are projected to experience similar demographic profiles in the coming decades. China is also undergoing rapid demographic aging. Its latest seventh national population census showed that the country has the largest number of older people in the world, with 260 million and 190 million above 60 and 65 years old, respectively [16]. Given China's large population size, even modest increases in the proportion of older adults will translate into substantial increases in the absolute number of individuals at risk of osteoporosis.

By 2075, individuals aged 50 years and above are projected to comprise over half of the population in several countries and regions in the Asia Pacific, including China (55%), Chinese Taipei (59%), Hong Kong SAR (55%), Japan (57%), the Republic of Korea (61%), Singapore (51%), and Thailand (52%). More alarming growth rates are predicted in countries like Nepal, Bhutan and Pakistan, which will see increases of 159%, 156% and 134% respectively in number of individuals aged above 50 years, reflecting a narrow window for early preventive strategies. The policy change opportunity is still open, but not indefinitely.

The shift is even more pronounced in the oldest age groups. By 2075, at least 30% of the population will be aged 70 years and above in China (30%), Chinese Taipei (35%), Hong Kong SAR (31%), Japan (33%), and Republic of Korea (37%), with dramatic increases seen in countries such as Mongolia (474%), Nepal (452%), and Bhutan (435%).

As a result, the incidence rates of fractures are projected to increase exponentially. In Singapore, osteoporotic fractures are expected to increase by 57.9%, rising from 15,267 cases in 2017 to 24,104 cases in 2035. Similarly, projections in Malaysia foresee a 3.55-fold increase in hip fractures, from 5,880 in 2018 to 20,893 by 2050. In Australia, over a decade from 2023 to 2033, the number of fragility fractures is expected to climb by 34%, reaching 237,632 by 2033. The cumulative total number of new and repeat fractures is expected to exceed 2.1 million cases in the same period.

These consistent trends signal the demographic transformation that will place sustained pressure on health systems. Without substantial improvements in preventive care, particularly for age-related conditions such as osteoporosis, healthcare services risk being overwhelmed by the growing burden of fragility fractures and associated long-term care needs.

At the same time, low fertility rates of less than 1.0 in countries like Korea and Singapore have resulted in an inverted population pyramid, increasing the dependency ratio [17]. With a shrinking caregiving base, and concentrated health spending in older age groups, osteoporosis will carry profound socio-economic burden. In Singapore, the total economic burden associated with fragility fractures is estimated to grow 50% from 2017 to 2035 [18].

These figures are not merely demographic curiosities; they represent future fracture caseloads, rehabilitation needs and medication demands, as well as long-term care pressure. An aging society is our inevitable expected future, but without timely preparations, our society and healthcare systems will be in crisis. This audit conveys several insights and a call for action.

### 3.2 Insights and call for action

#### 3.2.1 The power of numbers: Fracture surveillance provides impetus for change

Robust fracture surveillance and registries are fundamental prerequisites for effective strategic planning. Yet, the APCO-IOF regional audit found that only 10 of the 22 countries and regions maintain centralized fracture databases. They include Australia, Chinese Taipei, Hong Kong SAR, Japan, New Zealand, Pakistan, Philippines, Republic of Korea, Singapore, and Thailand. Most of the centralized databases (7 out of 10) are organized at the national level. Meanwhile, Philippines organizes its database at the regional level, and Hong Kong SAR and Thailand manage them at the hospital level.

Only eight countries and regions collect annual data on hip fractures, and only a quarter (6 out of 22) systematically record all fracture types. Despite so, the rates of patient record entry per capita varies greatly. This ranges from 2,510 per million population in Singapore to 8,325 per million in the Republic of Korea. Japan records 363,000 fractures surgically fixed yearly including 170,000 hip fractures. This is despite a surveillance gap that still exists for non-surgically managed fractures in Japan. Meanwhile, the database in Thailand only documents 110 patients with fractures per year. This is likely due to the databases being managed at an individual hospital level.

For the remaining countries, fracture incidence is estimated from fragmented datasets. This matters because policy attention follows visibility. Without reliable registries of fracture incidence, mortality, refracture rates, and treatment initiation, policy makers are blinded to the true magnitude of osteoporosis burden. Osteoporosis therefore competes poorly for budget, workforce, and infrastructure. This delays necessary policy responses and allocation of appropriate healthcare workforce and infrastructure, leaving societies unprepared for the growing burden of osteoporosis. For example, countries like Indonesia and India do not have centralized databases, and osteoporosis is not yet recognized as a healthcare priority.

#### 3.2.2 Recognition of osteoporosis as a healthcare priority

Despite demographic trends that clearly position osteoporosis as an urgent healthcare priority, it remains under-recognized at the policy level across much of the Asia Pacific region.

Only 6 out of 22 countries (Australia, China, Japan, New Zealand, Singapore and Thailand) explicitly recognize osteoporosis as a national health priority. This single statistic explains much of the downstream variation in osteoporosis care observed across the region. Health systems that formally prioritize osteoporosis are more likely to have explicit strategies, funded models of care, guideline uptake, registries and quality standards. In contrast, systems that do not formally prioritize osteoporosis are more likely to remain fragmented.

China offers an instructive example of what formal recognition can enable. Since osteoporosis was designated as a national health priority in 2022, greater emphasis has been placed on access to osteoporosis diagnostics and treatments, both of which are reimbursed through the National Health System. These efforts are closely aligned with public health initiatives, such as the *China Health Initiative* (2017–2025) which promotes nutritional support (specifically vitamin D and calcium intake), physical activity, fall prevention strategies, early screening, and comprehensive post-fracture management under the *‘Healthy Bone Action’* framework. Its most recent *Healthy China Action Plan* (2019–2030) also reiterated disease prevention through a nationwide campaign. In parallel, community support and patient engagement have continued to expand.

Without policy prioritization, it is difficult to develop coordinated national strategies, or to mobilize funding and infrastructure support towards improving osteoporosis care. First, budget allocation frameworks are typically tied to nationally recognized priorities. In the face of competing national demands, such as diabetes mellitus and infectious disease prevention, both of which often have established targets and performance indicators, osteoporosis remains structurally disadvantaged within national health agendas. Secondly, workforce planning is affected: without policy recognition, osteoporosis is rarely embedded into training curricula or primary care, reinforcing the specialist-driven care and, reactive condition rather than a managed chronic disease.

Importantly, absence of prioritization also limits the integration of osteoporosis into existing health system platforms. Unlike cardiovascular disease or diabetes mellitus, osteoporosis is often excluded from chronic disease programs, screening initiatives, and digital health infrastructure. This results in missed opportunities to leverage established systems for risk identification, monitoring, and long-term care.

Finally, the absence of national prioritization reduces data visibility and policy feedback mechanisms. Without registries and routine outcome tracking, fracture incidence and outcome measures remain poorly recognized. This creates a self-reinforcing cycle: low visibility leads to low prioritization, which in turn perpetuates underinvestment and fragmented care. Breaking this cycle requires explicit policy recognition, which serves as the foundational trigger for coordinated strategy, sustainable financing, and measurable system performance.

Robust policies tailored to the anticipated changes in population dynamics must be implemented early before a larger greying tsunami hits.

#### 3.2.3 Building the foundation: National guidelines and quality indicators

Guidelines are fundamental in translating evidence into clinical care. In the Asia Pacific region, 16 of 22 countries and regions have established osteoporosis guidelines.

However, even when guidelines exist, their presence alone is insufficient to ensure high-quality care. The audit found substantial variation in in their scope, methodological rigor, and frequency of updates. Across the region, national standards for osteoporosis management can be broadly categorized into three levels of system maturity.

The most advanced frameworks are seen in Australia, Japan, New Zealand, and Thailand, where national guidelines incorporate defined key performance indicators, which turn guidance into accountability. Regular public reporting promotes systematic benchmarking of provider performance and enables assessment of whether patients are actually receiving evidence-based care.

Australia's Hip Fracture Clinical Care Standard is a strong example of this principle. The Australian Commission on Safety and Quality in Health Care (ACSQHC) released the second edition of the Hip Fracture Clinical Care Standard in 2023, emphasizing timely surgical intervention, early mobilization, and strategies to reduce the risk of subsequent fractures [19]. Compliance with the standard is benchmarked and publicly reported through the Annual Reports of the Australian and New Zealand Hip Fracture Registry (ANZHFR), promoting continuous quality improvement [20]. Thailand has most recently joined this group. Quality indicators including rates of hip fracture surgery, subdivided into those performed within 72 h hours, and FLS referrals are tracked annually [21]. As data collection for this program began in July 2025, no data is available at the present moment for analysis.

The second group of countries and regions, including Bhutan, China, Chinese Taipei, Hong Kong SAR, Indonesia, Mongolia, Myanmar, Nepal, the Philippines, and Republic of Korea, have included some quality indicators in their clinical guidelines. However, data reporting is less frequent and systems for audit and benchmarking appear less well established.

In contrast, the third group, comprising Bangladesh, Brunei Darussalam, India, Malaysia, Pakistan, Singapore, Sri Lanka, and Vietnam, currently do not have formal national quality standards for fragility fracture care. Without clear guidance, osteoporosis care is more likely to be fragmented and variable, depending heavily on individual physician. An imperative step towards improving osteoporosis care would be the introduction of basic quality indicators, alongside regional collaboration and support.

Ultimately, guidelines provide the blueprint, but it is the consistent measurement of performance that ensures the blueprint is delivered and our society grows in the care of osteoporosis. When quality indicators are clearly defined, systematically audited, and, where appropriate, publicly reported, they create accountability and enable benchmarking across health systems. Key metrics such as time to hip fracture surgery, postoperative mortality, and rates of post-fracture osteoporosis treatment initiation provide objective assessment. Timely hip fracture surgery within 48 is associated with reduced mortality and complications [22]. Recently proposed WHO benchmarks for equitable hip fracture care and osteoporosis treatment in older adults further reinforce the importance of measurable system-level indicators in evaluating and improving fragility fracture care globally [23].

The audit highlights stark disparities in surgical timelines across the region. In high-income, system-mature countries such as Australia, Japan, and New Zealand, time to surgery is monitored as a national quality indicator.

Benchmarks are enforced through registries, and performance is publicly reported. For instance, the Japanese Ministry of Health, Labor and Welfare introduced a reimbursement policy in April 2022 incentivizing early surgery within 48 h. This policy was implemented in response to the projected increase in the incidence of hip fractures from 240,000 in 2020 to 320,000 by 2040 [24].

In contrast, in many low- and middle-income countries, time to surgery is not routinely tracked and delays may extend to several days or longer. A combination of reasons contributes: i) Limited operating theatre capacity, ii) Manpower constraints, iii) Financial barriers, iv) Delays in pre-operative optimization, and v) Fragmented referral pathways. Even when surgical capacity exists, systemic inefficiencies such as delayed diagnosis, lack of prioritization, or administrative bottlenecks, can significantly prolong time to intervention.

Embedding such measurable standards within clinical pathways, aligning them with reimbursement and policy frameworks, and ensuring regular audit and feedback mechanisms are essential steps towards improving outcomes in osteoporosis care.

#### 3.2.4 Diagnostic availability and therapeutic advancements do not translate to access

Dual-energy X-ray absorptiometry (DXA) remains the gold standard for the diagnosis of osteoporosis and fracture risk stratification [25,26]. However, the audit found that the availability of DXA does not consistently translate into meaningful access across the Asia Pacific region, and in many settings, it functions as a diagnostic bottleneck that delays treatment initiation.

In Brunei Darussalam, only one DXA scanner is available in the tertiary hospital, with access restricted to hospital physicians. This is similar in Bhutan, where there is only one axial DXA scanner located in a single tertiary center. Even when DXA is available, waiting times vary greatly, ranging from same-day access in some private centers to up to one year in public healthcare systems, particularly in settings where DXA is reimbursed, such as Hong Kong SAR and Malaysia.

This disparity reflects a broader structural imbalance. Public sector facilities, which manage the majority of patients, often face high demand and limited capacity, whereas private sector services offer more rapid access but at substantially higher cost. As a result, patients who rely on publicly funded care may experience significant delays in diagnosis, while those able to afford private services benefit from almost immediate assessment.

Cost further amplifies inequity. The audit reports a wide variation in DXA pricing, from approximately USD $15 in Vietnam to USD $175 or more in the Philippines. Even when absolute costs appear modest, they may represent a substantial financial burden in lower-income settings.

Reimbursement policies also vary considerably. In some healthcare systems, such as Japan, DXA is fully reimbursed with minimal or no waiting time, highlighting how aligned financing and infrastructure can enable efficient access. In contrast, in settings where DXA is publicly funded but capacity is constrained, such as Hong Kong SAR, despite nominally free access, long waiting times persist. In countries like Bangladesh, Indonesia and Philippines, limited or absent reimbursement places the full cost burden on patients, further restricting access.

Geographic distribution adds another layer of inequity. In many countries, DXA scanners are concentrated in urban centers, leaving rural populations with limited or no access to diagnostic services. Even in countries where machines are technically available, overall capacity may be insufficient due to low scanner density, shortages of trained personnel, or restricted operating hours.

Importantly, the audit shows that conventional metrics such as the number of DXA machines per capita are insufficient indicators of system readiness. A more meaningful concept is functional access, defined by timely availability, affordability, and effective linkage to treatment. Delayed access to DXA not only postpones diagnosis and treatment initiation, but may also compromise its utility for monitoring treatment response, particularly in high-risk individuals requiring timely intervention.

In settings where access remains limited, clinicians may rely on clinical risk assessment tools or initiate treatment without DXA confirmation in high-risk patients. While pragmatic, such approaches inherently compromise quality osteoporosis care, without timely objective assessments. Expanding equitable access to DXA, aligning reimbursement policies, and integrating diagnostic pathways with treatment initiation are therefore critical priorities for improving osteoporosis care across the Asia Pacific region.

#### 3.2.5 Risk assessment tools

Over the years, osteoporosis care has shifted to a greater emphasis on risk-based rather than bone mineral density (BMD)-based treatment decision-making. Fracture risk assessment tools are increasingly incorporated into osteoporosis guidelines, with 20 of 22 countries and regions in the Asia Pacific utilizing at least one fracture risk assessment tool. Mongolia and Vietnam do not use risk assessment tools. The most commonly used tools were FRAX® and the Garvan fracture risk calculator.

However, the audit also highlights several important limitations:

1. Risk assessment tools are designed to inform clinical judgement; they do not, in isolation, initiate care. A calculated probability that does not influence referral, treatment eligibility, or follow-up is informational but not transformative. Risk assessment tools must be integrated into actionable items.
2. Reimbursement alignment is crucial. Risk assessment tools are often disconnected from reimbursement systems, being a mere theoretical risk classification, and depriving some high-risk patients of potentially more efficacious medications.
3. FRAX® is the most commonly used risk assessment tool in 90% of the countries in Asia Pacific. Because healthcare costs, competing mortality risks, and fracture epidemiology vary substantially across the diverse health systems of Asia Pacific, FRAX® requires local calibration and country-specific intervention thresholds that better reflect national epidemiology and health-economic context.
4. The uptake of FRAX Plus®, which was introduced to refine fracture risk estimation, remains limited, with fewer than 20% of countries incorporating it into practice. This is in large part due to the fees associated with its use.

Ultimately, the audit emphasizes that risk assessment must be locally calibrated and integrated into diagnostic and treatment pathways linked to reimbursement criteria, if it is to fulfill its potential in improving osteoporosis care.

#### 3.2.6 Fractures beget fractures: The role of Fracture Liaison Services

FLS represent one of the most effective and rigorously validated model for secondary fracture prevention, yet adoption across the Asia Pacific remains patchy and inconsistent [27]. The multidisciplinary pathway links orthopedic surgeons with osteoporosis specialists and primary care, converting sentinel fragility fractures into a not-to-be-missed opportunity for timely diagnosis, treatment initiation, and longitudinal follow-up. The evidence is unequivocal: FLS programs improve treatment rates and adherence, reduce refracture risk, mortality, and healthcare costs [28]. For instance, in Thailand, implementation of an FLS program increased DXA testing rates from 28% to 86% and treatment rates from 41% to 89%, while reducing secondary fractures by 30% within one year. FLS also consistently reduces overall healthcare costs despite increased expenses for interval reviews and secondary fracture prevention [29].

Despite this strong evidence base, the overall averages of identifying fractures through FLS were dismal at 27%, 18% and 13% for hip, vertebral and other fractures, respectively across all countries and regions in the Asia Pacific. A quarter of the countries in the audit report no FLS, and in over half, coverage is limited to less than 25 percent of hospitals. This is an important finding, because it shows that although FLS has gained broad conceptual acceptance, it has not yet been normalized as standard of care across the region.

Even where FLS exists, they are often implemented as short-term pilot initiatives without integration into standard clinical practices. Post-fracture treatment uptake across the region presently remains highly variable, ranging from < 20% to nearly 90%, reflecting profound inequities in care.

Fragmented and short-term funding remains a fundamental barrier to the sustainability of FLS. Many FLS programs across the Asia Pacific region operate on precarious financial models, reliant on hospital budgets, research funding, or philanthropic support. Such arrangements are inherently unstable and incompatible with the sustained, system-wide impact that FLS are designed to achieve. Without sustainable funding, even well-performing programs risk eventual failure, limiting their reach and continuity of care. The long-term success of FLS eventually requires formal national commissioning frameworks with clearly defined referral triggers, and national health system funding linked to measurable outcomes.

Singapore is identified as one of the few countries in the Asia Pacific region with relatively advanced implementation of FLS, with more than 50% of hospitals reported to have an established FLS. FLS in Singapore operate within a mixed funding model and are integrated into broader osteoporosis care frameworks that include fracture identification, risk assessment, treatment initiation, and long-term follow-up [30]. While the audit focuses primarily on system-level availability and coverage, experience from established FLS programs in Singapore and elsewhere has highlighted persistent challenges in treatment initiation, adherence, and long-term persistence [31]. These observations emphasize that beyond implementation, the effectiveness of FLS depends on sustained patient engagement and continuity of care, and that mature programs can serve as important learning platforms for other countries in the region seeking to scale and strengthen secondary fracture prevention services.

A leading example of integrated, registry-enabled secondary fracture prevention in the Asia Pacific is New Zealand where two complementary registries drive systematic data collection to improve treatment outcomes following fragility fractures: the Australian and New Zealand Hip Fracture Registry (ANZHFR) and the Australian and New Zealand Fragility Fracture Registry (ANZFFR), established in 2015 and 2022 respectively [32,33]. The ANZHFR registry benchmarks care across the different phases of hip fracture management: pre-operative, operative, post-operative, and rehabilitation, tracking outcomes up to 120 days post-fracture. It monitors adherence to the binational Hip Fracture Care Clinical Care Standard, which was updated in 2023 [19]. All hospitals in the nation have an FLS and all hip fractures are referred to an FLS. In 2024, by 120-day follow-up, 54% of hip fracture patients were receiving osteoporosis-specific therapy (eg, bisphosphonates, denosumab, or teriparatide), while a further 30% were receiving calcium and vitamin D supplementation alone [34]. The highest-performing hospitals achieved osteoporosis-specific therapy rates of 84% and 79%, providing an important benchmark for the national FLS quality improvement program as it seeks to raise performance more uniformly across the country [34]. Beyond hip fractures, the ANZFFR also addresses secondary fracture prevention across all fragility fractures. Within just two years, the registry scaled markedly to achieve one of the fastest rates of patient identification globally, capturing 55% of the estimated fracture caseload in its first year and 72% in its second year. This data-driven approach has translated into improved clinical outcomes. Within 12 weeks of fracture, 99% of patients received fracture risk assessment, 96.5% falls risk assessment, and 85% received education on bone health [35]. By 16 weeks, 90% had follow-up care, and 82% had a documented long-term care plan communicated to primary care [35]. At one year, refracture rates were only 3.3%, alongside high treatment adherence [35]. Together, these registries demonstrate how integrated surveillance, defined quality indicators, and continuous audit can produce an accountable, high-performing national system.

Preventing the next predictable fracture is imperative. Scaling FLS is therefore not optional, but essential, as it offers one of the most immediate, measurable, and cost-effective strategies to reduce the burden of recurrent fractures at a population level.

#### 3.2.7 A sustainable future for the healthcare system

Osteoporosis care across the Asia Pacific region remains highly specialist-driven, a model that is increasingly unsustainable in the face of rapidly aging populations, as specialist services become bottlenecks. Fifteen out of 22 countries and regions reported that osteoporosis is not primarily under the care of primary care physicians. Orthopedic surgeons were involved in osteoporosis care in nearly all settings, while endocrinologists and rheumatologists were also commonly involved. This pattern highlights a structural issue at the heart of regional preparedness: if osteoporosis care remains concentrated within specialist services, many health systems will struggle to deliver timely preventive care at population scale. In countries such as Indonesia and Malaysia, reimbursement criteria and prescribing rights are limited for primary care physicians. Such constraints inherently delay diagnosis and shift the point of intervention downstream, often to the time of fragility fracture rather than earlier risk identification and prevention.

The seven countries and regions where osteoporosis is primarily under the care of primary care physicians are: Australia, Hong Kong SAR, Japan, Mongolia, New Zealand, Singapore, and Vietnam. Japan provides an important example of how this model can function in practice. There, osteoporosis is largely managed by primary care physicians, while specialist input is provided by orthopedic surgeons, gynecologists, endocrinologists, and geriatricians. This model reflects the recognition that specialist-led care alone cannot meet the demands of an aging population. It is not a matter of whether primary care should replace specialists, but how responsibility can be appropriately distributed. Specialist-driven models may remain appropriate for complex or very high-risk patients, but they are poorly suited to meet the demands of the whole population. Population aging will increase the number of individuals requiring fracture risk assessment, and osteoporosis medications. These tasks cannot be managed efficiently if each step depends on specialist entry.

Although the audit indicates that osteoporosis is recognized as a medical specialty in some countries and is incorporated into specialty training in others, there must be broad-based competence rather than narrow expertise alone. Primary care physicians need confidence in identifying high-risk patients, initiating first-line therapy, interpreting DXA findings, using fracture risk calculators, and referring appropriately. Orthopedic teams need pathways that trigger bone health evaluation after fracture, while nurses and allied health professionals require defined roles in patient education, falls prevention, and follow-up. This requires investment in education, formulation of clear clinical pathways, and alignment of reimbursement and prescribing policies.

Ultimately, osteoporosis must transition from a specialist-managed condition to a system-wide responsibility. Hong Kong SAR offers an example of such a model, where osteoporosis is managed across the system by primary care physicians, internal medicine physicians, endocrinologists, rheumatologists, geriatricians, and orthopedic surgeons. It also forms a key component of specialty medical training, particularly in endocrinology and rheumatology. Endocrinologists and rheumatologists manage the majority of complex and very high-risk cases. Similarly, in China, osteoporosis care is delivered through both primary and specialist services, with patients able to seek care in either setting. These examples are important because they show that effective osteoporosis care does not depend on removing specialists from the pathway, but on distributing responsibility more rationally across the health system. A mature model of care requires not just specialists, nurses, and coordinators, but also primary care be in involved in both primary and secondary fracture prevention.

#### 3.2.8 Osteoporosis awareness and advocacy in the community

In many settings across the Asia Pacific region, public awareness of osteoporosis and fragility fractures remains low. As a result, individuals at risk may not seek screening or preventive care until a fracture occurs. Patient advocacy organizations can contribute in ways that clinical systems often cannot: they promote awareness before fracture occurs, provide patient support through the disease journey, and help elevate osteoporosis as a legitimate public health concern [36]. However, the audit revealed that patient advocacy organizations exist in just nine countries and regions - Australia, Hong Kong SAR, Indonesia, Japan, Malaysia, Mongolia, New Zealand, Singapore, and Thailand.

Indonesia provides an important example through PERWATUSI (Perhimpunan Warga Tulang Sehat Indonesia; Indonesian Healthy Bones Society), a national patient support organization dedicated exclusively to osteoporosis. PERWATUSI is particularly active in outreach initiatives, including partnerships with community and women's organizations. It also developed a tailored exercise program, *Senam Osteoporosis*, which targets not only older adults but also younger populations as part of a prevention strategy. Close collaboration is also forged by PERWATUSI with PEROSI (Perhimpunan Osteoporosis Indonesia, the Indonesian Osteoporosis Association), a professional medical society, forming the bridge between evidence-based clinical guidance and patient support.

Patient support efforts are also encouraging in Australia. Healthy Bones Australia, a national not-for-profit organization was established in 2001 in response to the rising prevalence of osteoporosis and limited awareness of the disease. It is involved in policy development, education, peer support, and research and development.

In China, while there are no osteoporosis-specific patient support organizations, several foundations and non-profit organizations run osteoporosis initiatives that target the general public. This includes education lecture series such as “Strengthen muscles and bones, prevent and treat osteoporosis” (强肌健骨,防治骨松), and national drug-assistance programs like “Bone-Dance Life” (骨舞人生-严重骨质疏松患者援助项目) which provides financial aid for the use of teriparatide in patients with severe osteoporosis. These examples show that osteoporosis awareness and patient support can still be advanced even in the absence of a single disease-specific advocacy body.

Digital technology is also emerging as a useful tool for community engagement. In addition, mobile telehealth platforms in the form of WeChat, are increasingly being used for patient engagement with positive effects in enhancing knowledge in China [37]. Beyond its use for patient education and follow-up support, WeChat has been used to also help residents locate nearby osteoporosis diagnosis and treatment centers, improving access to convenient care.

Public awareness matters at multiple points along the osteoporosis pathway: from primary and secondary fracture prevention to treatment adherence and fall prevention. To effectively reach the wider population, national osteoporosis strategies should therefore incorporate public awareness campaigns and educational programs, supported by technology to enhance accessibility and engagement. Such efforts should be regarded not as optional additions to clinical care, but as foundational components of a comprehensive fracture prevention strategy.

#### 3.2.9 Reimbursement determines reality

Access to effective pharmacologic therapy greatly influences osteoporosis outcomes, yet across the Asia Pacific region, substantial disparities exist in both treatment availability and reimbursement. These disparities are not fully explained by whether a medicine is available in a country. Rather, real-world access is determined by whether therapies are affordable, reimbursed, and hence accessible across routine clinical settings. In this sense, reimbursement determines reality.

These findings highlight two distinct access problems. The first is non-reimbursement, where patients must bear a significant, if not the full cost of treatment out of pocket. Treatment options are often also limited, also influenced by availability and costs. For instance, reimbursements for osteoporosis medications are not provided in certain countries, substantially limiting access to treatment. Calcium and vitamin D supplementation are still designated as first-line therapies in many countries in the Asia Pacific and osteoporosis medication availability is limited to bisphosphonates in several others. Even with the limited range of medications, reimbursement is conditional on specific criteria including age, BMD and secondary prevention. This compounded by a lack of national osteoporosis guidelines, no central DXA services, and no fracture risk assessment tools, further constrains osteoporosis care in several countries. Within the public healthcare system, some patients may receive subsidized or free treatment based on financial need; however, access and quality of services vary widely by region. Non-governmental organizations may help bridge these gaps by providing additional financial support. In the private sector, most patients bear the cost of treatment, with partial assistance through private health insurance if enrolled.

The second issue is limited reimbursement for newer more effective therapies, with a misalignment between clinical guidance and reimbursement policies thus constraining personalized treatment recommendations. Oral bisphosphonates are universally accessible in all countries and regions in the Asia Pacific, with reimbursement for alendronate in 18 of 22 countries and regions. Thirteen of 22 countries and regions offer reimbursement for zoledronate. While bisphosphonates remain effective for many patients, exclusive reliance on these agents limits the ability to implement risk-stratified treatment strategies, particularly for individuals at highest risk of imminent fracture.

The evidence for anabolic agents as first-line therapy in individuals at very high fracture risk is overwhelming and consistent [38]. However, only three countries in the region (Australia, China, and India), formally designate anabolic therapies as first-line options in their treatment guidelines. Teriparatide is reimbursed in only eight countries, romosozumab in six, and abaloparatide in just one.

In India, the 2021 Indian Society for Bone and Mineral Research (ISBMR) guidelines support a risk-stratified approach, including the use of anabolic therapy as initial treatment in selected high-risk patients [39]. However, access to such therapies is constrained by cost and lack of reimbursement. Reimbursement is commonly available in public hospitals but is infrequent in the private sector. More than 60% of healthcare in India is delivered through the private sector. While approximately 40% of the population has some form of health insurance, the coverage is often limited and typically excludes routine outpatient care, where osteoporosis therapy is initiated. As a result, treatment remains an out-of-pocket expense for most patients. Furthermore, reimbursement is also generally applicable to generic medications rather than newer drugs. While romosozumab has been launched in India since 2024, it is not widely used due to the high cost. All other osteoporosis therapies are available as generic or biosimilar drugs manufactured locally in India, which helps reduce costs.

In certain well-resourced systems with access to the full spectrum of therapies and comprehensive reimbursement frameworks, reimbursement policies frequently lag behind clinical evidence and constrain personalized clinical recommendations. This disconnect often occurs as guidelines are developed by professional societies and non-governmental organizations, while government authorities independently make reimbursement decisions. Guidelines might endorse an anabolic-first approach for very high-risk patients, yet reimbursement requires prior failure of bisphosphonates, creating a misalignment between evidence-based care and policy implementation. Anabolics like teriparatide and romosozumab are not subsidized in China.

Strict reimbursement criteria may also be tied to BMD thresholds, further restricting access. In Chinese Taipei, where antiresorptive medications are reimbursed as first-line therapy, while reimbursement for anabolic agents applies only to patients at very high fracture risk, defined by a BMD T-score of ≤ −3.0, who have already received antiresorptive therapy for more than one year and sustained a new fragility fracture. Nevertheless, policy progress has been made in recent years. Since 2025, reimbursements have extended beyond hip and vertebral fractures to also include radius and humerus fractures. Recognition and reimbursement are also given to high-risk factors for osteoporosis, including a history of rheumatoid arthritis, diabetes mellitus on insulin therapy, and high dose steroid use. Medications are covered almost fully by the National Health Insurance system with very limited co-pay when patients fit the reimbursement criteria.

Limiting access to effective treatment does not reduce costs, it shifts them downstream. Preventable fractures result in increased healthcare utilization, loss of independence, and broader socioeconomic burden, including caregiver strain and reduced productivity. The short-term cost savings associated with non-reimbursement, or restricting access to higher-cost therapies must be weighed against the long-term economic and societal costs of untreated osteoporosis. Japan offers one example of a system in which broad reimbursement may support more individualized care. Under the national health system, osteoporosis treatment is reimbursed in full, without a single designated first-line therapy, and reimbursement is linked instead to criteria such as DXA findings and indication for secondary fracture prevention. Similarly, Australia has a robust and comprehensive reimbursement system through its national Pharmaceutical Benefits Scheme (PBS). The system aims to support both primary and secondary prevention of fragility fractures. This includes individuals with prior fragility fractures, those with low BMD and aged 70 years or older or on prolonged high dose glucocorticoid therapy.

Ultimately, the audit highlights that therapeutic innovation alone is insufficient. The benefits of newer osteoporosis treatments can only be realized if they are accessible to the populations that need them most. Reimbursement frameworks must be aligned with clinical risk stratification. Sustainable pricing models and international collaboration may be necessary to improve access in lower-income settings. Without such alignment, therapeutic innovation will continue to be distributed inequitably across the Asia Pacific region.

## 4 The way forward

Despite significant challenges, the Asia Pacific region has come a long way in its osteoporosis care. There is a rapidly expanding FLS coverage, national guidelines, established diagnostic and therapeutic capabilities in many countries. Regionally, strong networks are forged through professional bodies such as APCO, IOF and Asian Federation of Osteoporosis Societies (AFOS).

Nevertheless, the APCO-IOF Asia Pacific Regional Audit has brought to attention the marked heterogeneity in osteoporosis care in the region. The audit proposed a roadmap of ten pragmatic action priorities.

1. Formal designation of osteoporosis as a national health priority.
  • Formal prioritization of osteoporosis is the enabling step that brings osteoporosis into planning, financing, and accountability systems. Epidemiological studies must be started in countries where the current disease burden is not known, to bring awareness to the weight of this problem.
2. Establishment and expansion of fracture registries.
  • Countries without national fracture registries should begin with a pragmatic minimum dataset centered on hip fractures, which are clinically identifiable. From there, systems can expand toward broader fragility-fracture capture, linkage to mortality and treatment data, and integration with FLS or rehabilitation pathways.
3. Universal access to best-practice post-fracture care, including orthogeriatric services and FLS.
  • Every fragility fracture should trigger a secondary prevention pathway. In practical terms, this means making FLS part of standard processes.
4. Promotion of lifestyle interventions in nutrition and exercise, tailored to various patient profiles
  • Early prevention for children and adolescents: National osteoporosis societies to collaborate with education bodies, nutrition and sports councils, to promote healthy eating and physical activities to allow children and adolescents achieve their peak bone mass.
  • Adults and seniors: National osteoporosis societies should partner with government ministries responsible for aging, nutrition, sport and recreation. These collaborations should aim to educate individuals on appropriate nutrition and physical activity to prevent early bone and muscle loss, prevent malnutrition and falls in the elderly.
5. Strengthening of professional education across relevant specialties.
  • National osteoporosis societies and healthcare professional organizations should work closely to develop and promote broad participation in structured education programs tailored to different groups, including specialists, primary care physicians, allied health professionals, and nursing staff.
6. Implementation of routine fracture risk assessment in primary care for adults aged 50 years or older.
  • This can be achieved through close collaboration between national osteoporosis societies and primary care physicians.
7. Integration of osteoporosis management into prescribing practice.
  • High quality evidence should be integrated into clinical guidelines and decision-making.
8. Development of public awareness initiatives.
  • Prevention cannot rely solely on clinical encounters after fractures occur. National osteoporosis societies should actively promote public awareness of osteoporosis through educational initiatives, encouraging ownership of citizens in the care of osteoporosis, and patient advocacy.
9. Creation of national alliances for falls and fragility fracture prevention.
  • In countries without a national alliance, osteoporosis societies should engage with relevant stakeholders such as policymakers and non-governmental organizations, to establish a coordinated falls and fracture prevention alliance, drawing on successful international models.
10. Scaling of digital and data-driven innovations to improve surveillance, equity, and efficiency.
  • Digitalization and artificial intelligence present a world of opportunities. National osteoporosis societies, policymakers, and healthcare professional organizations should work together to pilot and expand innovative strategies (eg, telehealth, virtual rehabilitation services) that improve efficiency, accessibility, and equity in fracture prevention.
  • The central conclusion of the APCO-IOF Asia Pacific Regional Audit is not that osteoporosis care in the region is failing everywhere. Rather, it is that the region is facing many warning signs of a future crisis. Demographic change is already underway. The biology of osteoporosis is well understood. Effective diagnostic tools and therapies are widely available. The challenge is no longer scientific, but rather structural.

Health systems must now decide whether osteoporosis will remain a peripheral concern or be recognized as a central test of their capacity to respond to population aging. The data are clear, the tools are available, and the blueprint has been articulated.

The question is no longer whether action is possible, but whether inaction is acceptable.

## CRediT author statement

**Hui Min Charlotte Choo:** Conceptualization, Writing – Original draft. **Manju Chandran:** Conceptualization, Writing – Original draft, Writing – Review & editing. **Paul Mitchell, Peter R Ebeling, Philippe Halbout, Nicholas C Harvey, Eugene V McCloskey, Ambrish Mithal:** Conceptualization, Writing – Review & editing.

## Conflicts of interest

Hui Min Charlotte Choo declares no competing interests.

Manju Chandran chairs APCO and is on the Scientific Advisory Board of APCO and is on the Board of IOF. Paul Mitchell is on the Scientific Advisory Board of APCO and is owner of Synthesis Medical NZ Ltd, New Zealand.

Peter R Ebeling is on the Scientific Advisory Board of APCO and on the Board of IOF. Philippe Halbout is on the Scientific Advisory Board of IOF and is the chief executive officer of IOF. Nicholas C Harvey is the President of IOF. Eugene V McCloskey is Chair, Council of Scientific Advisors of the IOF. Ambrish Mithal is on the Scientific Advisory Board of APCO and on the Board of IOF.

## References

[1] International database. https://www.census.gov/data-tools/demo/idb/#/dashboard?dashboard_page=country&COUNTRY_YR_ANIM=2026.

[2] Kanis J.A., Johnell O., Oden A., Dawson A., De Laet C., Jonsson B. (2001). Ten year probabilities of osteoporotic fractures according to BMD and diagnostic thresholds. Osteoporos Int.

[3] Johnell O., Kanis J.A., Odén A., Sernbo I., Redlund-Johnell I., Petterson C. (2004). Fracture risk following an osteoporotic fracture. Osteoporos Int.

[4] (2025). The APCO-IOF Asia Pacific regional audit: epidemiology, costs and burden of osteoporosis in 2025. International Osteoporosis Foundation.

[5] (1993). Consensus development conference: diagnosis, prophylaxis, and treatment of osteoporosis. Am J Med.

[6] Curtis E.M., van der Velde R., Moon R.J., van den Bergh J.P.W., Geusens P., de Vries F. (2016). Epidemiology of fractures in the United Kingdom 1988–2012: variation with age, sex, geography, ethnicity and socioeconomic status. Bone.

[7] Kanis J.A., Johnell O., Oden A., Sernbo I., Redlund-Johnell I., Dawson A. (2000). Long-term risk of osteoporotic fracture in malmö. Osteoporos Int.

[8] Chandran M., Brind'Amour K., Fujiwara S., Ha Y.C., Tang H., Hwang J.S. (2023). Prevalence of osteoporosis and incidence of related fractures in developed economies in the Asia Pacific region: a systematic review. Osteoporos Int.

[9] Alajlouni D.A., Alarkawi D., Tran T.S., Prior J.C., Kovacs C.S., Berger C. (2026). Fractures at virtually all sites are associated with adverse outcomes: an observational study. J Bone Miner Res.

[10] Shen Y., Huang X., Wu J., Lin X., Zhou X., Zhu Z. (2022). The global burden of osteoporosis, low bone mass, and its related fracture in 204 countries and territories, 1990-2019. Front Endocrinol.

[11] Autier P., Haentjens P., Bentin J., Baillon J.M., Grivegnée A.R., Closon M.C. (2000). Costs induced by hip fractures: a prospective controlled study in Belgium. Osteoporos Int.

[12] Schousboe J.T. (2016). Epidemiology of vertebral fractures. J Clin Densitom.

[13] Tosteson A.N.A., Gabriel S.E., Grove M.R., Moncur M.M., Kneeland T.S., Melton L.J. (2001). Impact of hip and vertebral fractures on quality-adjusted life years. Osteoporos Int.

[14] Hatano M., Yasunaga H., Ishikura H., Tanaka T., Aso S., Tanaka S. (2025). Temporal trends in acute care costs of hip fracture treatment from 2011 to 2021 in Japan. Arch Osteoporosis.

[15] Melton I.I.I.L.J., Crowson C.S., O'Fallon W.M. (1999). Fracture incidence in olmsted county, Minnesota: comparison of urban with rural rates and changes in urban rates over time. Osteoporos Int.

[16] Akimov A.V., Gemueva K.A., Semenova N.K. (2021). The seventh population census in the PRC: results and prospects of the country's demographic development. Herald Russ Acad Sci.

[17] Kim J.K. (2024). The predetermined future: tackling South Korea's total fertility rate crisis. Clin Exp Pediatr.

[18] Chandran M., Lau T.C., Gagnon-Arpin I., Dobrescu A., Li W., Leung M.Y.M. (2019). The health and economic burden of osteoporotic fractures in Singapore and the potential impact of increasing treatment rates through more pharmacological options. Arch Osteoporosis.

[19] Australian Commission on Safety and Quality in Health Care (2023).

[20] Australian and New Zealand Hip Fracture Registry (September 2025). Annual report of hip fracture care.

[21] Thailand: surveillance and prevention system for falls, hip fractures and recurrent fractures | capture the fracture. https://www.capturethefracture.org/news/thailand-surveillance-and-prevention-system-falls-hip-fractures-and-recurrent-fractures.

[22] Klestil T., Röder C., Stotter C., Winkler B., Nehrer S., Lutz M. (2018). Impact of timing of surgery in elderly hip fracture patients: a systematic review and meta-analysis. Sci Rep.

[23] Chandran M., Thiyagarajan J.A., Alokail M., Bruyère O., Harvey N.C., Rizzoli R. (2026). WHO benchmarks for equitable hip-fracture care and osteoporosis treatment in older people. Nat Rev Rheumatol.

[24] Yamamoto N., Sawaguchi T., Matsushita T., Katoh N., Arai H., Shirahama M. (2024). Fragility fracture network-Japan: the challenge of establishment of a national hip fracture database and successful achievement of nationwide health system change for hip fracture care in Japan. Injury.

[25] Marshall D., Johnell O., Wedel H. (1996). Meta-analysis of how well measures of bone mineral density predict occurrence of osteoporotic fractures. BMJ.

[26] Johnell O., Kanis J.A., Oden A., Johansson H., De Laet C., Delmas P. (2005). Predictive value of BMD for hip and other fractures. J Bone Miner Res.

[27] Pinedo‐Villanueva R., Burn E., Maronga C., Cooper C., Javaid M.K. (2023 Apr 1). Expected benefits and budget impact from a microsimulation model support the prioritization and implementation of fracture liaison services. J Bone Miner Res.

[28] Chandran M., Akesson K. (2013). Secondary fracture prevention: plucking the low hanging fruit. Ann Acad Med Singapore.

[29] Kobayashi S., Tanaka S., Yoshino Y., Tobita H., Kuwagaki K., Fujioka R. (2022). Impact of osteoporosis liaison services on the expected lifetime osteoporosis-related medical expenses of patients with fragility fracture in a private hospital in Japan. Arch Osteoporosis.

[30] Chandran M., Tan M.Z.W., Cheen M., Tan S.B., Leong M., Lau T.C. (2013). Secondary prevention of osteoporotic fractures—an “OPTIMAL” model of care from Singapore. Osteoporos Int.

[31] Chandran M., Cheen M., Ying H., Lau T.C., Tan M. (2016). Dropping the ball and falling off the care wagon. Factors correlating with nonadherence to secondary fracture prevention programs. J Clin Densitom.

[32] Zhou Y., Wall C.J., Hallen J., Mitchell R., Taylor M.E., Harvey L.A. (31 Oct 2025). Data resource profile: Australian and New Zealand Hip Fracture Registry (ANZHFR). HIP Int.

[33] Australian & New Zealand Fragility Fracture Registry (April 2026). https://www.fragilityfracture.co.nz.

[34] Australian and New Zealand Hip Fracture Registry (September 2024).

[35] Australian & New Zealand Fragility Fracture Registry (April 2025). Annual report of fragility fracture registry data 2025. https://fragilityfracture.co.nz/2025-annual-report/.

[36] Chen Y., Zhang Y., Zheng Q., Sun L. (2025). Lived experiences and insights of Chinese patients with symptomatic osteoporosis on a patient-reported outcome (PRO) programme: a qualitative phenomenological study in Southwest China. BMJ Open.

[37] She P., Yang J., Xu L., Xiong Y., Zhang Z., Wu Z. (2022). The effects of WeChat official account based osteoporosis education for patients with fragility fracture in China. Int J Orthop Trauma Nurs.

[38] Chandran M. (2022). The why and how of sequential and combination therapy in osteoporosis. A review of the current evidence. Arch Endocrinol Metab.

[39] Nagendra L., Bhavani N., Menon V.U., Pavithran P.V., Menon A.S., Abraham N. (2021). FRAX-based osteoporosis treatment guidelines for resource-poor settings in India. Arch Osteoporosis.

